# ﻿*Yushaniatomentosa* (Poaceae, Bambusoideae), a new combination from Guangxi

**DOI:** 10.3897/phytokeys.218.97312

**Published:** 2023-01-10

**Authors:** Xing Li, Jing-Bo Ni, Fei Tan, Yi-Hua Tong, Nian-He Xia

**Affiliations:** 1 Key Laboratory of Plant Resources Conservation and Sustainable Utilization & Guangdong Provincial Key Laboratory of Applied Botany, South China Botanical Garden, Chinese Academy of Sciences, CN-510650, Guangzhou, China South China Botanical Garden, Chinese Academy of Sciences Guangzhou China; 2 South China National Botanical Garden, CN-510650, Guangzhou, China South China National Botanical Garden Guangzhou China; 3 University of Chinese Academy of Sciences, CN-100049, Beijing, China University of Chinese Academy of Sciences Beijing China; 4 The Administration Bureau of Jiuwan Mountain National Natural Reserve, CN-545300, Rongshui, China The Administration Bureau of Jiuwan Mountain National Natural Reserve Rongshui China

**Keywords:** bamboo, Jiuwan Mountain, *Sasa*, taxonomy

## Abstract

*Sasatomentosa* is transferred to the genus *Yushania* following a reassessment based on a new collection with pachymorph and long-necked rhizomes from its type locality in Guangxi, China. Morphologically, it is most similar to *Yushaniadoupengshanensis*, but differs in culm, branch complement and foliage leaf characters. A revised description of its morphology and color photos are also provided.

## ﻿Introduction

*Sasa* Makino & Shibata (1901) with 43 species (Soreng et al. 2022) distributed in East Asia, is characterized by leptomorph rhizomes, solitary branches at each node, panicle-like synflorescences, and six stamens and three stigmas per floret. The results of previous molecular phylogenetic analyses showed that *Sasa* is polyphyletic with species dispersed in several clades or subclades (Triplett and Clark 2010; Zeng et al. 2010; Zhang et al. 2012). In China, eight species (all endemic) were recognized under *Sasa*, of which only *S.sinica* was reported based on flowering material (Keng 1936; Hu 1985, 1996; Wang and Stapleton 2006). Hu (1985) divided the Chinese *Sasa* into two subgenera, i.e., S.subg.Sasa and S.subg.Sasamorpha (Nakai) C. H. Hu. Based on newly collected flowering material, Qin et al. (2021) described a new genus, *Sinosasa* L. C. Chia ex N. H. Xia, Q. M. Qin & Y. H. Tong, to accommodate some species of S.subg.Sasa from China with raceme-like synflorescences, two to three florets per spikelet with a rudimentary terminal floret, three stamens and two stigmas per floret, strongly raised culm supranodal ridges, and relatively long (> 1 cm) foliage leaf ligules. Hitherto, seven species are recognized in this new genus, including three previously recognized species of S.subg.Sasa and four new species. However, due to the limitations of sampling and time, not all the species of *Sasa* from China were studied by Qin et al. (2021), and the taxonomic position of some species without raised supranodal ridges on culm and very long foliage leaf inner ligules was still uncertain. More recently, a little-known *Sasa* species from China, *S.guangdongensis* W. T. Lin, was synonymized with *Acidosasacarinata* (W. T. Lin) D. Z. Li & Y. X. Zhang based on morphological considerations (Li et al. 2022).

**Figure 1.**
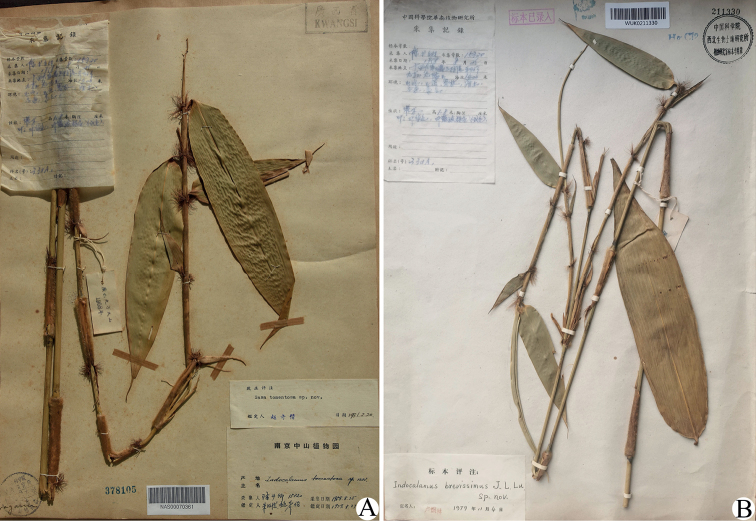
Isotypes of *Sasatomentosa* (*S. H. Chun 15320*, **A** NAS00070361 **B** WUK0211330). Photo **A** by Wei Zhou, **B** by Liang Zhao.

*Sasatomentosa* C. D. Chu & C. S. Chao was described in 1981 based on two specimens of the only collection of *S. H. Chun 15320* from Jiuwan Mountain in Guangxi, China (Chao and Chu 1981), with the one in IBK designated as the holotype and the other in NAS as the isotype. We have not found the holotype yet, although an attempt to search for it in IBK was made. The line drawing of *S.tomentosa* in the protologue is obviously based on the isotype in NAS (Fig. 1A), as they are nearly the same. Judging from the line drawing in the protologue or the isotype in NAS, it is hard to determine which genus this species should be assigned to, because the specimen does not have a rhizome and only contains a very young culm before branching development, while both rhizome and branch complement are key characters to differentiate genera of bamboos. Fortunately, we found another three duplicates of the collection *S. H. Chun 15320* deposited in IFP, N and WUK. The isotype deposited in WUK (Fig. 1B) possesses two branches at a node of culm, which conflicts with the strictly solitary branch at each culm node of *Sasa*. Thus, *S.tomentosa* should not belong to *Sasa*, although the treatment of its assignment to *Sasa* has been accepted by many floras, such as Flora Reipublicae Popularis Sinicae, Flora of China and Flora of Guangxi (Hu 1996; Wang and Stapleton 2006; Xia et al. 2016) over the past 40 years. This species has neither strongly raised culm supranodal ridges nor long foliage leaf ligules, so it should not be assigned to *Sinosasa* either. Thus, the taxonomic position of this species needs further study.

## ﻿Materials and methods

The complete specimens, including rhizome, culm, branches and leaves of *S.tomentosa*, were collected from the type locality, viz., Jiuwan Mountain National Natural Reserve, Rongshui County, Guangxi Zhuang Autonomous Region, China during a field trip in September 2022. Observations and measurements were taken using a magnifier and a ruler with the scale of 0.5 mm. Some minor characters such as indumentum on ligules of both culm leaves and foliage leaves were observed with a stereomicroscope (Mshot MZ101). The description was made based on both living and dried material and also consultation with the relevant literature (Chao and Chu 1981; Hu 1996; Wang and Stapleton 2006; Huang and Dai 2009; Xia et al. 2016). Herbarium acronyms follow Thiers (2022).

## ﻿Results and discussion

**Figure 2.**
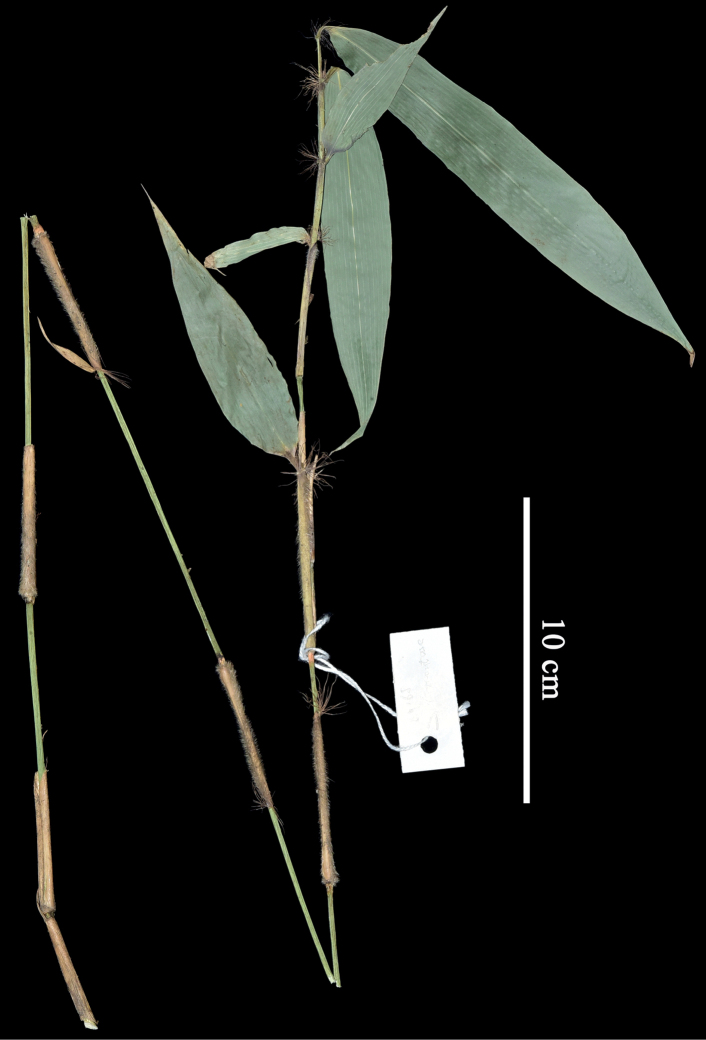
Specimen of *Yushaniatomentosa*, *X. Li & J. B. Ni LX168* (IBSC).

**Figure 3.**
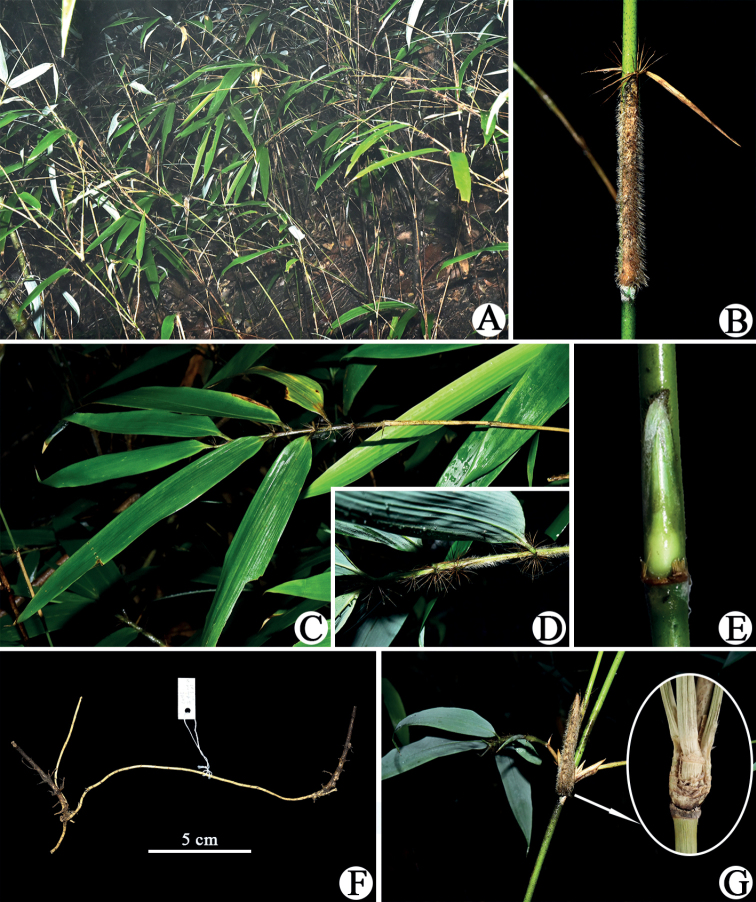
*Yushaniatomentosa***A** habit **B** culm leaf and partial culm **C** foliage leaf branch **D** part of foliage leafy branch, showing tomentose sheath and radiate oral setae **E** culm bud **F** pachymorph rhizome with long neck **G** three branches at an upper culm node. All photos by Xing Li.

When the first and second authors visited the type locality of *Sasatomentosa* in September 2022, only two bamboo species were found, i.e., *Chimonobambusaangustifolia* C. D. Chu & C. S. Chao and one *Yushania* species. The young culm without branches of the latter bamboo species (Fig. 2) that we collected matched the isotype in NAS (Fig. 1A) very well, and shares the same key characters, such as the abaxially densely hirsute culm leaf sheath (Fig. 3B), the falcate auricles, the radiate oral setae and the short ligules of culm leaves, the glabrous internodes, the slightly prominent nodes, the white powdery infranodal region, and the culm sheath 1/2–1/3 as long as the internode, as described in the protologue. Thus, we are certain that the specimens we collected are *S.tomentosa*.

However, except for the characters mentioned above, some of its other characters fit well with the circumscription of *Yushania* Keng f. (Keng 1957) rather than *Sasa*, such as pachymorph rhizome with long necks (Fig. 3F), the branch complement with mostly solitary branch at lower culm nodes, one to two at mid and upper culm nodes and rarely three at upper culm nodes (Fig. 3G) and persistent culm leaf sheaths. Thus, *Sasatomentosa* is obviously a member of the genus *Yushania*.

Chao and Chu (1981) perhaps misunderstood the young culm as an ultimate leafy branch, because they described the ultimate branches with two to three wavy foliage leaves when dried in the protologue. In fact, this bamboo has ultimate branches with four to eight much smaller foliage leaves than those described by Chao & Chu.

After examining the specimens of similar species and referring to the related literature (Yi 1986; Li et al. 2006; Zhang and Xia 2021), it is found that *S.tomentosa* is most similar to *Yushaniadoupengshanensis* Y. Y. Zhang et N. H. Xia (Zhang and Xia 2021) in sharing several vegetative characters, such as falcate auricles, radiate oral setae and truncate ligules of culm and foliage leaves, reflexed and lanceolate culm leaf blades, glabrous foliage leaf blades and white puberulous pseudopetioles, but differs in having nearly solid (vs. hollow) culms, branch complements with usually solitary branch at lower culm nodes, one to two at mid and upper culm nodes and rarely three at upper culm nodes (vs. solitary at each culm node), glabrous (vs. densely white puberulous) internodes with (vs. without) dense purple spots, and densely hirsute (vs. sparsely setose) foliage leaf sheath with ciliate (vs. glabrous) margins. A more detailed comparison between the two species is provided in Table 1. Thus, it is confirmed that *S.tomentosa* represents a distinct species of *Yushania*. Accordingly, a new combination of *S.tomentosa* under *Yushania* is made as follows.

**Table 1.** Comparison of *Yushaniatomentosa* and *Y.doupengshanensis*.

Characters	*Y.tomentosa*	*Y.doupengshanensis*
Culm internode	Nearly solid, 15–27 cm long, glabrous, green and densely purple-spotted, not powdery except infranodal region	Hollow, 14–18 cm long, densely white puberulous, green without purple spots, thinly white powdery and densely so on infranodal region
Branch complement	Usually 1 at the basal culm nodes, 1 to 2 at mid and upper culm nodes, rarely 3 at upper culm nodes	Solitary at each culm node
Culm leaf auricle	Falcate, 2–5 × 1–2 mm	Broadly falcate, 2.5‒3 × 1‒1.5 mm
Abaxial surface of foliage leaf sheath	Densely hirsute	Sparsely setose
Margin of foliage leaf sheaths	Ciliate with trichomes readily deciduous	Glabrous
Type locality	Guangxi (Rongshui County)	Guizhou (Duyun County)

### ﻿Taxonomic treatment

#### ﻿ Yushania tomentosa

Taxon classificationPlantaePoalesPoaceae

(C.D.Chao & C.S.Chao) N.H.Xia, Y.H.Tong, J.B.Ni & X.Li comb. nov.

0AD6F98E-84F3-596E-89E0-A3B878B71DB3

urn:lsid:ipni.org:names:77311667-1

Figs 1 , 2 , 3

Sasa tomentosa C. D. Chu & C. S. Chao, J. Nanjing Technol. Coll. Forest Prod. 3(3): 35 (1981). Basionym.

##### Type.

China. Guangxi: Rongshui County, Jiuwan Mountain, elev. 1400 m, 25 August 1958, *S. H. Chun 15320* (holotype: IBK, not seen; isotypes: NAS00070361, image!; WUK0211330, image!; N019023167, image!; IFP15899999w0005, image!).

##### Description.

Shrubby bamboo. Rhizomes pachymorph, necks 20–30 cm long, 3–5 mm in diameter, solid. Culms 1–2 m tall, 2–6 mm in diameter, diffuse; branches intravaginal, developing from 4^th^ to 5^th^ nodes upwards, usually solitary at lower culm nodes, 1–2 at mid and upper culm nodes and rarely 3 at upper culm nodes; internodes terete, 15–27 cm long, glabrous, densely purple-spotted, thickly white powdery below nodes, nearly solid; nodes slightly prominent; supranodal ridges flat or slightly raised; intranodes 3–5 mm long, glabrous. Culm buds solitary, long-ovate to lanceolate, yellow to light green, ciliate on the margin, apex attenuate, base obtuse. Culm leaf sheaths persistent, 1/2–1/3 as long as internodes, densely white to yellowish-brown hirsute with trichomes 3–5 mm long, densely ciliate on the margin; sheath scar prominent, with persistent remains of sheath base; auricles falcate, 2–5 × 1–2 mm; oral setae developed, radiate, 8‒12 mm long; ligule truncate, 0.5–1 mm high, ciliolate on the margin; blades linear-lanceolate to lanceolate, 1.2–3.5 × 0.2–0.7 cm, reflexed, ca. 1/2 as long as culm sheath, glabrous, margin serrulate. Foliage leaves 4–8 per ultimate branch; sheath densely white hirsute with trichomes ca. 2 mm long, margin ciliate, sometimes glabrescent, longitudinal ribs conspicuous; auricles falcate, 1–3 × 0.5–1.5 mm; oral setae radiate, 10‒13 mm long; inner ligule ca. 1 mm high, truncate or oblique-truncate, ciliolate on the margin; outer ligule ca. 0.5 mm high, margin white ciliate with trichomes 0.5‒1 mm long; pseudopetioles white puberulous, initially white powdery, 5‒8 mm long; blades long-lanceolate to lanceolate, 13–22 × 1.5–2.6 cm, wavy when dry, glabrous, apex long-acuminate, base cuneate to obtuse, margin sparsely serrulate or sometimes entire; secondary veins 7–9 pairs, transverse veins conspicuous. Inflorescence unknown.

##### Phenology.

New shoots from August to September.

##### Vernacular name.

Róng Máo Yù Shān Zhú (Chinese pronunciation); 绒毛玉山竹 (Chinese name).

##### Additional specimens examined.

*Yushaniatomentosa*: China. Guangxi: Rongshui County, Wangdong Township, Jiuwan Mountain, Weilinjiang, 23 September 2022, 25°18'39.3"N, 108°38'13.2"E, elev. 1358 m, *X. Li & J. B. Ni LX168* (IBSC). *Yushaniadoupengshanensis*: China. Guizhou: Duyun County, Doupeng Mountain, 29 November 2020, 26°22'39"N, 107°21'25"E, elev. 1200 m, *Y. Y. Zhang 2004* (holotype: IBSC0865924!; isotypes: IBSC0865925!, IBSC0865926!).

## Supplementary Material

XML Treatment for Yushania tomentosa
